# Aortic intimal sarcoma mimicking a mycotic pararenal abdominal aortic aneurysm

**DOI:** 10.1016/j.jvscit.2025.101832

**Published:** 2025-05-08

**Authors:** Kentaro Kasa, Takao Ohki, Kota Shukuzawa, Hirotsugu Ozawa, Takeshi Baba, Masayuki Hara

**Affiliations:** Division of Vascular Surgery, Department of Surgery, The Jikei University School of Medicine, Tokyo, Japan

**Keywords:** Aortic intimal sarcoma, Superior mesenteric artery stenosis, Tumor embolism

## Abstract

We present a case of aortic intimal sarcoma mimicking a mycotic para-renal abdominal aortic aneurysm, treated with staged hybrid surgery. A 60-year-old female presented with complaints of nausea and back pain. Computed tomography showed a para-renal abdominal aortic aneurysm with irregular and multilobulated shape, without periaortic soft-tissue inflammation, occlusion of the celiac artery, and stenosis of the superior mesenteric artery. She underwent superior mesenteric artery stenting, followed by aortic resection and axillo-femoral bypass for suspicion of mycotic aortic aneurysm. Pathologic examination revealed aortic intimal sarcoma. Two years after surgery, she died of multiple organ failure due to sepsis during chemotherapy for bone metastases.

Aortic intimal sarcoma (AIS) is a very rare tumor with clinical presentations and conventional imaging findings that are often nonspecific. Preoperative diagnosis of AIS is challenging because it frequently presents with atypical courses or findings compared with other aortic diseases, such as aneurysms. Herein, we present a case of AIS mimicking a mycotic para-renal abdominal aortic aneurysm (PRAAA), treated with staged hybrid surgery.

## Case report

Written consent was obtained from the patient for the publication of the details and images of this case.

**Fig 1:**
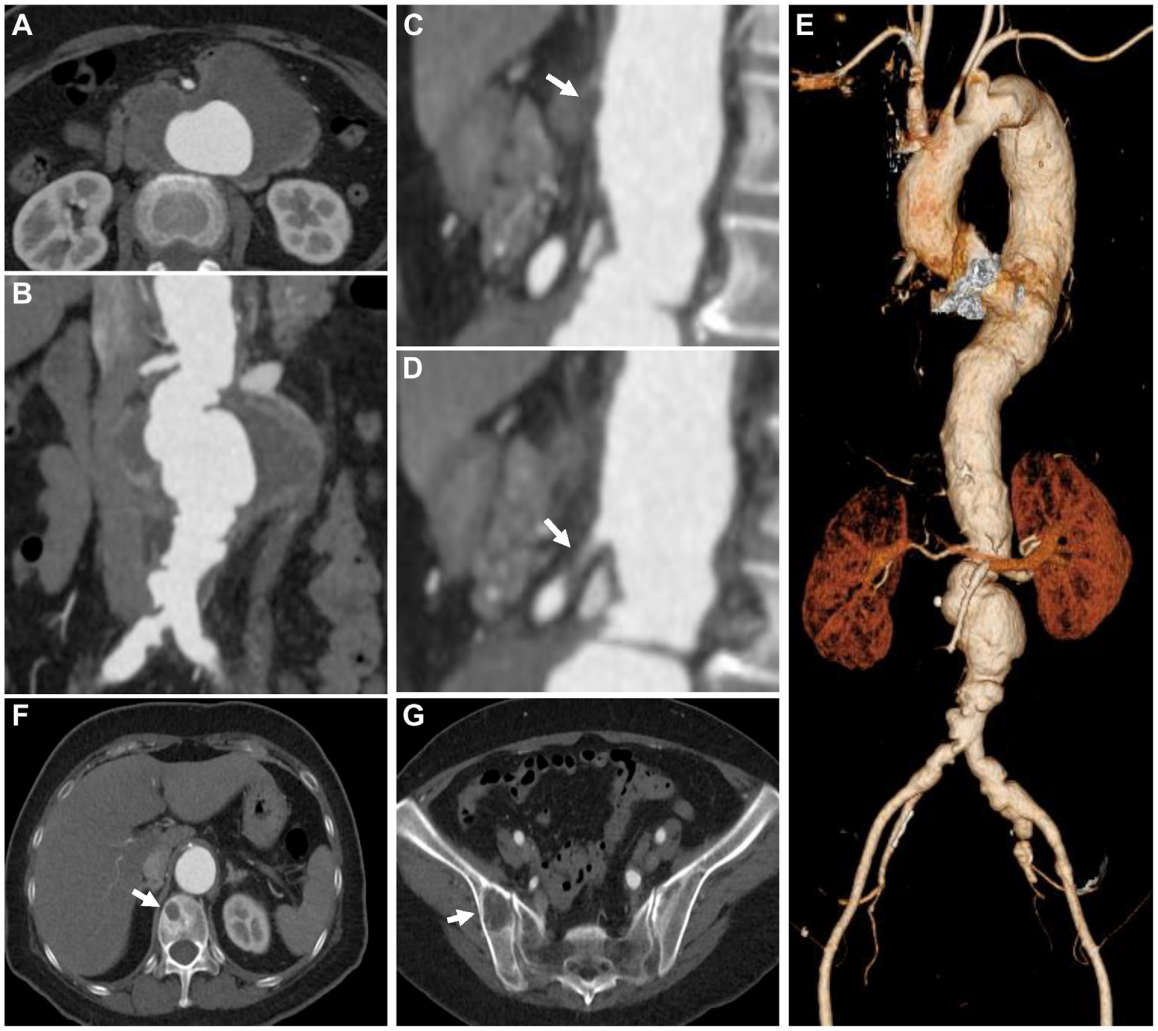
Preoperative computed tomography (CT). **(A)** Irregular and lobulated para-renal abdominal aortic aneurysm (PRAAA) with irregular and multilobulated shape, without periaortic soft-tissue density and inflammation surrounding the aneurysm on the axial image. **(B)** PRAAA on the coronal image. **(C)** Occluded celiac artery (CA) (*arrow*) on the sagittal image. **(D)** Stenotic superior mesenteric artery (SMA) (*arrow*) on the sagittal image. **(E)** Three-dimensional reconstruction of the aorta. **(F)** Osteolytic changes in the 12th thoracic vertebra (*arrow*) on the axial image. **(G)** Mass in the iliac bone (*arrow*) on the axial image.

A 60-year-old female with a history of hypertension, dyslipidemia, Hashimoto’s thyroiditis, gastroesophageal reflux disease, and uterine fibroids visited her primary care physician with a chief complaint of nausea and back pain. She was referred to our hospital with a suspected mycotic aortic aneurysm, based on blood tests showing elevated inflammatory markers and computed tomography (CT) findings of high-attenuation soft tissue shadows around the abdominal aorta. She had no history of smoking and no family history of aortic disease. On physical examination, she had a height of 158 cm, a weight of 60 kg, a low-grade fever, and a pulsatile abdominal mass with mild tenderness. Laboratory tests revealed an elevated white blood cell count and serum C-reactive protein level (9500/μL and 14.45 mg/dL, respectively). Repeat blood cultures taken on admission were negative. Contrast-enhanced CT showed a PRAAA with irregular and multilobulated shape, without periaortic soft-tissue inflammation, occlusion of the celiac artery (CA) and the inferior mesenteric artery, and severe stenosis of the superior mesenteric artery (SMA) (Fig 1, *A-E*). In addition, masses were observed in the 12th thoracic vertebra and the right iliac bone (Fig 1, *F* and *G*). [(67)Ga] scintigraphy showed uptake in the abdominal aorta from the CA branches to the aortic bifurcation. With a suspicion of a mycotic aortic aneurysm, conservative treatment was initiated. Given that the CA was already occluded preoperatively, the SMA was considered the primary source of blood supply to a wide range of abdominal organs, including those perfused by the CA. Because SMA occlusion during the waiting period prior to definitive surgery could have been fatal, we planned a staged hybrid approach, consisting of elective intervention for SMA stenosis, followed by secondary aortic resection and reconstruction.

### Operative procedure

**Fig 2:**
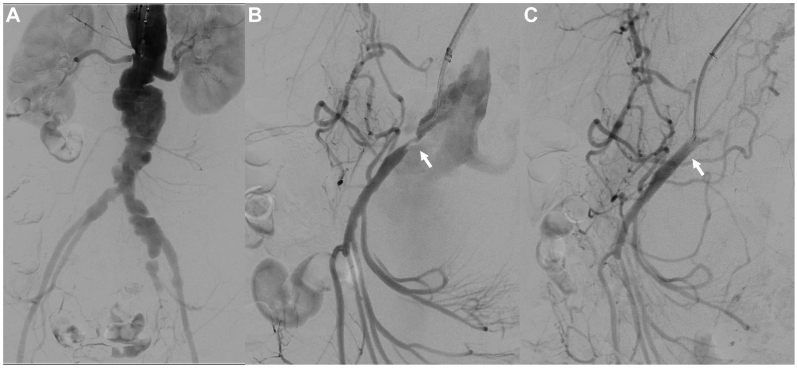
Intraoperative angiography of the initial surgery for superior mesenteric artery (SMA) stenosis. **(A)** Aortogram. **(B)** Selective angiogram showing stenosis at the origin of the SMA (*arrow*). **(C)** Postoperative selective angiogram demonstrating relief of SMA stenosis (*arrow*).

**Fig 3:**
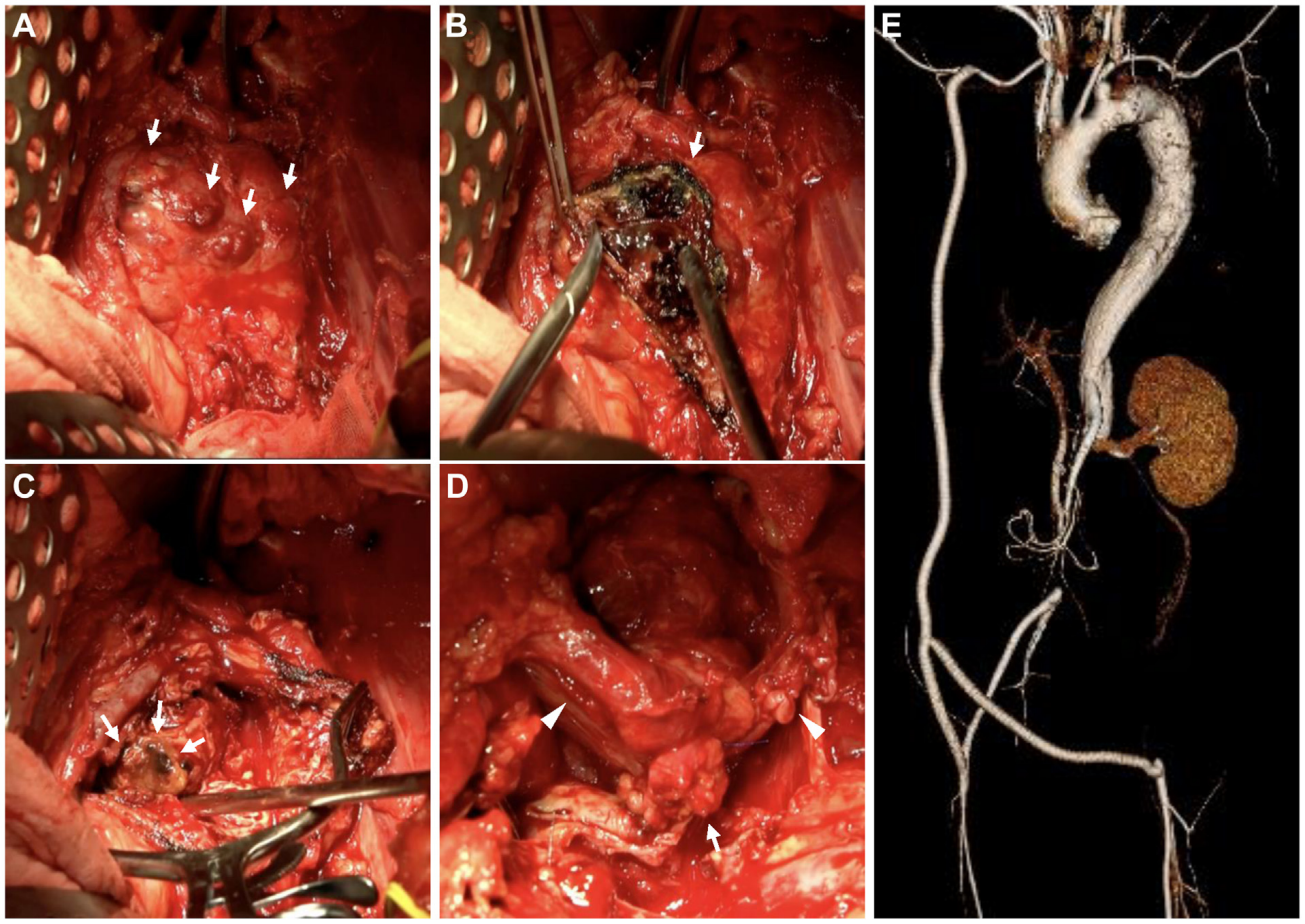
Intraoperative images. **(A)** Para-renal abdominal aortic aneurysm (PRAAA) with a multilocular aneurysmal wall (*arrows*). **(B)** Opening the aneurysm revealed partially mucinous contents. **(C)** Aorto-duodenal fistula (*arrows*). **(D)** Abdominal aortic stump just below the bilateral renal arteries (*arrowheads*). **(E)** Postoperative computed tomography (CT) showing a three-dimensional reconstruction of the aorta.

On the fifth day of hospitalization, direct stenting was performed via a right brachial artery approach to treat stenosis at the origin of the SMA using a 7 × 27 mm bare-metal stent (Express LD; Boston Scientific) (Fig 2). On the 12th day of hospitalization, definitive surgery was performed. First, an axillo-femoral bypass was completed. The PRAAA was exposed via a retroperitoneal approach, revealing a multilocular aneurysmal wall (Fig 3, *A*). Under aortic clamping proximal to the bilateral renal arteries, the aneurysm was opened, revealing partially mucinous contents (Fig 3, *B*). Examination from the luminal side of the aorta identified an aorto-duodenal fistula, which was closed with simple sutures (Fig 3, *C*). To facilitate maximal removal of the contents adjacent to the SMA, the aortic clamp was then repositioned proximal to the SMA, and the abdominal aorta was transected just below the bilateral renal arteries (Fig 3, *D*). The aneurysmal wall of the PRAAA and the left common iliac artery aneurysm were resected as extensively as possible, and the stumps of both iliac arteries were closed. The resected area was filled with omentum, and a gastrostomy and enterostomy were created. The surgery duration was 10 hours and 13 minutes, with an estimated blood loss of 4700 mL.

### Postoperative course

On the next day following surgery, the patient demonstrated elevated lactate levels on arterial blood gas analysis. Contrast-enhanced CT revealed significant in-stent restenosis of the SMA along with signs of intestinal hypoperfusion. These findings necessitated the placement of an additional stent and an exploratory laparotomy. The in-stent restenosis of the SMA was presumed to be due to mural thrombus and intraluminal debris displaced into the stent during the surgical procedure; therefore, an additional bare metal stent was deployed to restore adequate mesenteric perfusion. On postoperative day 19, an upper gastrointestinal contrast study was performed, after which the patient began oral intake. Following rehabilitation to address postoperative deconditioning, the patient was discharged home 2 months after surgery. Postoperative CT showed good bypass blood flow (Fig 3, *E*), and [(67)Ga] scintigraphy revealed no uptake in or around the resected aneurysm area.

**Fig 4:**
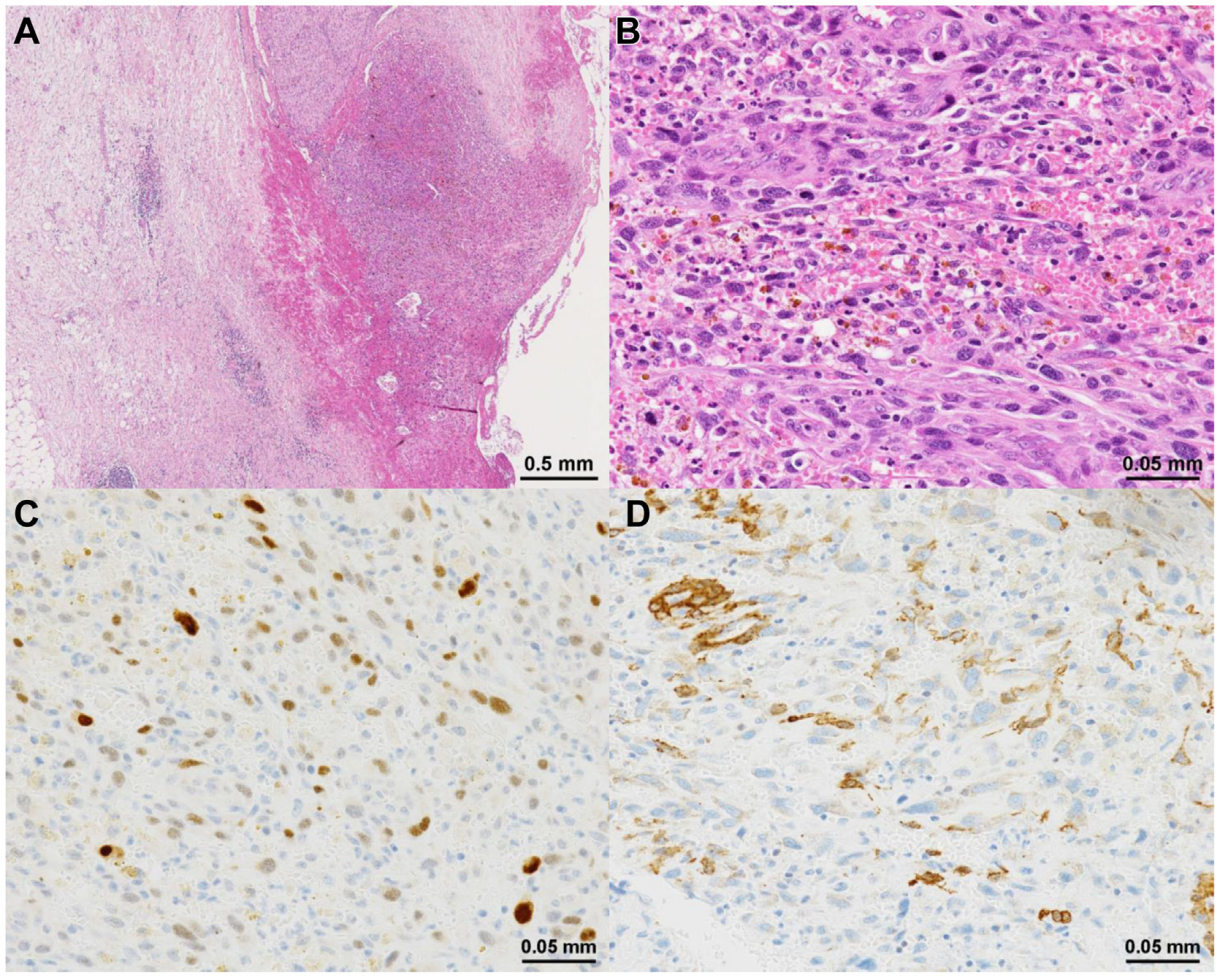
Pathologic findings of the resected aorta. **(A)** Indistinct structure of the vessel wall (hematoxylin and eosin stain). **(B)** Bundles or mat-like proliferation of atypical spindle-shaped cells (hematoxylin and eosin stain). **(C)** Focally positive expression for MDM2 of tumor cells. **(D)** Focal and weak positive expression for α-smooth muscle actin of tumor cells.

Cultures obtained from the resected specimens, including the aortic wall and surrounding retroperitoneal inflammatory tissues, were negative. Pathologically, the three-layer structure of the vessel wall (ie, intima, tunica media, and adventitia) was indistinct (Fig 4, *A*). In part of the aortic intima, bundles or mat-like proliferation of atypical spindle-shaped cells were observed (Fig 4, *B*). Immunohistochemically, the tumor cells were focally positive for MDM2 (Fig 4, *C*), focally and weakly positive for α-smooth muscle actin (Fig 4, *D*), equivocal for CDK4, and negative for desmin, CD68, CD31, CD34, and STAT6. Consequently, the diagnosis was most likely intimal sarcoma of the aorta.

The masses in the 12th thoracic vertebra and right iliac bone, noted on preoperative CT scan, were further evaluated with magnetic resonance imaging, which suggested a high likelihood of metastatic lesions. Chemotherapy was initiated 2.5 months postoperatively. At 15 months postoperatively, metastasis to the right adrenal gland was detected, and at 18 months, multiple bone metastases were identified, leading to changes in the chemotherapy regimen. At 17 months postoperatively, palliative radiotherapy was administered for the multiple bone metastases. Despite continuing chemotherapy, she died of multiple organ failure due to sepsis at 24 months postoperatively.

## Discussion

Intimal sarcomas are very rare malignant mesenchymal tumors that arise in large blood vessels of the systemic and pulmonary circulation, as well as in the heart.^1^, ^2^, ^3^ Pulmonary vessel sarcomas are twice as common as aortic tumors. The defining characteristic of these tumor is their predominantly intraluminal growth, leading to obstruction of the vessel lumen and embolic seeding to peripheral organs.^4^^,^^5^ AIS appears to be more common in males, with a mean age at diagnosis of 62 years.^1^, ^2^, ^3^ Most AIS cases originate in the abdominal aorta, typically between the CA and the iliac bifurcations, whereas 30% occur in the thoracic aorta.^2^^,^^3^^,^^5^ Clinical features and imaging findings are nonspecific, making preoperative diagnosis challenging. Due to its rarity, there are no established systemic treatments, such as chemotherapy or molecular targeted therapies, specifically for AIS.^6^ The prognosis for AIS is poor, with a mean survival time of 5 to 9 months.^7^

A review of the literature highlights the challenges in differentiating AIS from various other aortic diseases. Specifically, these include embolism,^8^^,^^9^ aortic stenosis,^10^ mycotic aneurysm,^11^ ruptured aneurysm,^12^^,^^13^ inflammatory aneurysm,^14^ aortitis,^15^ intramural hematoma,^16^ and stent graft infection.^17^ As indicated in these reports, preoperative diagnosis of AIS is extremely challenging. However, some studies have proposed the usefulness of fluorine-18-fluorodeoxyglucose positron emission tomography/computed tomography.^18^, ^19^, ^20^ fluorine-18-fluorodeoxyglucose positron emission tomography/computed tomography may help distinguish aortic sarcomas from mycotic aortic aneurysms because the standardized uptake value of sarcomas tends to be higher.

In the current case, AIS could have been suspected preoperatively based on three atypical CT findings:^1^ an aortic aneurysm with irregular and multilobulated shape, without periaortic soft-tissue density and inflammation surrounding the aneurysm,^2^ CA occlusion and SMA stenosis without findings of atherosclerosis,^3^ and masses in the 12th thoracic vertebra and right iliac bone. Based on the surgical findings, the CA and SMA lesions appeared to be caused by direct tumor invasion or external compression from the tumor, rather than tumor emboli. The lesions in the 12th thoracic vertebra and right iliac bone showed osteolytic changes, which were considered consistent with bone metastases. Taken together, these three findings suggest that AIS might have been considered preoperatively. As a supplementary note, the aorto-duodenal fistula identified during definitive surgery was subclinical and was not the primary factor of the current case.

There is no established management for AIS. Complete surgical resection of the tumor and surrounding tissues has been shown to prolong survival.^21^^,^^22^ A combination of surgical and medical treatments, particularly chemotherapy and radiotherapy, appear to have demonstrated the best survival outcome.^7^ In the current case, the patient’s prolonged survival was likely due to appropriate prior endovascular treatment for the SMA lesion, sufficient surgical resection (evidenced by the absence of uptake on postoperative gallium scintigraphy), and the initiation of chemotherapy relatively early after surgery.

## Conclusions

AIS is an extremely rare disease, and preoperative diagnosis is often challenging. Careful evaluation of atypical clinical courses and imaging findings may facilitate preoperative consideration of AIS, thereby leading to appropriate management for AIS.

## Funding

This work was supported by JSPS KAKENHI Grant Number JP24K11980.

## Disclosures

T.O. serves as a consultant to W.L. Gore and Associates.
